# Development and Characterization of Probe-Based Real Time Quantitative RT-PCR Assays for Detection and Serotyping of Foot-And-Mouth Disease Viruses Circulating in West Eurasia

**DOI:** 10.1371/journal.pone.0135559

**Published:** 2015-08-13

**Authors:** Syed M. Jamal, Graham J. Belsham

**Affiliations:** 1 Department of Biotechnology, University of Malakand, Chakdara, Dir (L), Khyber Pakhtunkhwa, Pakistan; 2 National Veterinary Institute, Technical University of Denmark, Lindholm, Kalvehave, Denmark; Ella Foundation, INDIA

## Abstract

Rapid and accurate diagnosis of foot-and-mouth disease (FMD) and virus serotyping are of paramount importance for control of this disease in endemic areas where vaccination is practiced. Ideally this virus characterization should be achieved without the need for virus amplification in cell culture. Due to the heterogeneity of FMD viruses (FMDVs) in different parts of the world, region specific diagnostic tests are required. In this study, hydrolysable probe-based real time reverse transcription quantitative polymerase chain reaction (RT-qPCR) assays were developed for specific detection and serotyping of the FMDVs currently circulating in West Eurasia. These assays were evaluated, in parallel with pan-FMDV diagnostic assays and earlier serotype-specific assays, using field samples originating from Pakistan and Afghanistan containing FMD viruses belonging to different sublineages of O-PanAsia, A-Iran05 and Asia-1 (Group-II and Group-VII (Sindh-08)). In addition, field samples from Iran and Bulgaria, containing FMDVs belonging to the O-PanAsia^ANT-10^ sublineage were also tested. Each of the three primer/probe sets was designed to be specific for just one of the serotypes O, A and Asia-1 of FMDV and detected the RNA from the target viruses with cycle threshold (C_T_) values comparable with those obtained with the serotype-independent pan-FMDV diagnostic assays. No cross-reactivity was observed in these assays between the heterotypic viruses circulating in the region. The assays reported here have higher diagnostic sensitivity (100% each for serotypes O and Asia-1, and 92% [95% CI = 81.4–100%] for serotype A positive samples) and specificity (100% each for serotypes O, A and Asia-1 positive samples) for the viruses currently circulating in West Eurasia compared to the serotyping assays reported earlier. Comparisons of the sequences of the primers and probes used in these assays and the corresponding regions of the circulating viruses provided explanations for the poor recognition of some of the viruses by the earlier assays. These new assays should help in the early detection and typing of serotype O, A and Asia-1 FMDVs circulating in West Eurasia to enable improved disease control.

## Introduction

Foot-and-mouth disease (FMD) is an infectious and highly contagious disease of cloven-hoofed domestic and wild animals, particularly cattle, buffalo, sheep, goats and pigs. The disease is caused by FMD virus (FMDV), belonging to the genus *Aphthovirus* within the family *Picornaviridae* [1]. FMDV is non-enveloped and has a single-stranded positive polarity RNA genome of about 8.3 kilobases. The virus exists in seven immunologically distinct serotypes: O, A, C, SAT (Southern African Territories) 1, SAT 2, SAT 3 and Asia-1. Multiple subtypes can be identified within each serotype that sometimes fail to induce good cross-protection against other viruses of the same serotype. The seven serotypes are not distributed uniformly around the world. Historically, the serotype O, A and C FMDVs have had the widest distribution globally and have been responsible for outbreaks in Europe, America, Asia and Africa but serotype C has not been detected anywhere since 2005 [2]. The serotype SAT 1, SAT 2 and SAT 3 viruses are normally confined to sub-Saharan Africa while serotype Asia-1 is restricted to Asia but occasional incursions of SAT serotypes into the Middle East/North Africa and of serotype Asia-1 into Europe have occurred [1].

FMD causes heavy economic losses to the livestock industry globally with an estimated annual cost of USD 6 to21 billion in agricultural damage and prevention expenditure [3] and has enormous potential impact on food security. The disease is endemic in many countries of Africa and Asia. In contrast, Europe, North America, Australia, New Zealand and some countries in Asia (e.g. Japan and South Korea) are normally free of FMD. However, outbreaks can spread from endemic areas to disease-free countries as occurred in recent years in the United Kingdom, France and The Netherlands in 2001 [4], Japan and Korea during 2000, 2010 and 2014, and Bulgaria during 2010–2011 [5–8].

Rapid and accurate diagnosis of FMD is of paramount importance for control of the disease. Various pan-FMDV reverse transcription quantitative polymerase chain reaction (RT-qPCR) assays for the detection of FMDV have been reported [9–12]. These assays target highly conserved RNA sequences either within the 5'-untranslated region (UTR) or in the sequences coding for non-structural proteins and they can efficiently detect each of the seven serotypes from clinical samples. These diagnostic assays possess high analytical sensitivity and specificity. Their analytical sensitivity has been shown to be at least equal to that of the current reference standard method, i.e. virus isolation in cell culture [12–14]. However, these assays do not determine the serotype of the particular FMDV that is detected. Identification of the serotype of the causative virus from outbreaks is important for vaccine selection, especially in endemic countries, for control of the disease. This is normally carried out using antigen detection ELISA but, as this test only has ca. 70% sensitivity (in clinical samples) [15], it is often necessary to propagate the virus in susceptible cells prior to this assay to establish the serotype and this clearly extends the duration of the procedure.

The VP1 is a surface exposed capsid protein that plays an important role in the antigenicity and pathogenicity of FMDV. It contains important immunogenic sites including amino acid residues within the G-H loop and the C-terminus. The G-H loop also includes an arginine-glycine-aspartic acid (RGD) motif, which is required for attachment of the virus to integrin receptors on the host cell [16,17]. Due to their heterogeneity, the nucleotide sequences encoding VP1 are extensively used for the determination of genetic relationships between different strains and for the tracing of the origin and movement of outbreak strains [18–23].

Conventional RT-PCR assays using primers targeting the VP1 coding region for serotyping of FMDV have been reported [24–26]. However, these assays either have relatively poor sensitivity and specificity, due to the genetic diversity within all FMDV serotypes, or are cumbersome and unsuitable for routine use [27,28]. Furthermore these assays are not sensitive enough to replace antigen detection and serotyping ELISA and virus isolation although they can be used as supportive tests [29,30]. Ideally, the serotyping should be performed without the need for virus amplification in cell culture.

Due to the heterogeneity of FMDVs circulating in different parts of the world, region-specific tests are required. Region-specific antigen detection ELISA and conventional RT-PCR assays for differentiation of FMDV serotypes, using multiple primers designed from nucleotide sequences of viruses circulating in that geographical area, have been developed [31]. These assays demonstrated the potential use of tailored molecular tools for the identification of serotype(s), within specific geographical regions, as an alternative to, or support for, the pan-FMDV RT-qPCR assays. Region-specific RT-qPCR assays have recently been reported for detection of SAT 2 (Group-VII) FMDVs circulating in East Africa [32] and for the serotyping of FMDVs circulating in the Middle East [33] and East Africa [34]. This study builds on the use of such systems as described previously.

Analyses of field samples from Pakistan and Afghanistan using primers and probes described by Reid and colleagues [33] showed that some viruses were not detected in these assays although they were positive in the pan-FMDV RT-qPCR assays described previously [10,12]. This failure in virus detection indicates the need to develop further RT-qPCR assays for typing of FMDVs (serotypes O, A and Asia-1) that circulate in West Eurasia. The present study was aimed at the development of such assays and to assess these assays in comparison with those described earlier [33]**.**

## Materials and Methods

### Design of serotype-specific primers and TaqMan probes

FMDV VP1 coding region sequences derived from viruses originating within the West Eurasian region were obtained from GenBank (www.ncbi.nlm.nih.gov). These sequences were aligned using MEGA 6 [35] and conserved regions were identified. Serotype-specific primers and probes for serotypes O, A and Asia-1 FMDVs were designed from the conserved sequences in the alignments (see Table 1).

**Table 1:** Sequences of primer and probe sets designed for serotyping of West Eurasian FMDVs using RT-qPCR assays.

Name of Primer/probe	Orientation	FMDV serotype	Sequence (5'– 3')
O-JB-F	Forward	O	GAGACAGCGTTGGAYAACACC
O-JB-R	Reverse	O	TGWGGTGCCGTGTAAGGCAG
O-JB-F-P	—-	O	Fam—AATCCAACGGCTTACCACAAGGCACC—Tamra
A-JB-F	Forward	A	GCCACGACCATCCACGAGCT
A-JB-R	Reverse	A	GTCCTGYGACRACACTTCCAC
A-JB-F-P	—-	A	Fam—CTCGTGCGYATGAAACGTGCYGAGCT—Tamra
As-JB-F	Forward	Asia-1	TGCCYACYTCXTTYAAYTACGG
As-JB-R	Reverse	Asia-1	CARAGGYCTRGGGCAGTATGT
As-JB-F-P	—-	Asia-1	Fam—CGTTTCATGCGRATYAAMAGCTCAGTGAT—Tamra

### Samples

Field samples (epithelial or oral swabs) originating from Pakistan and Afghanistan, containing FMDVs belonging to the lineages O-PanAsia, A-Iran05, A-Pak09 and Asia-1 (Groups II and VII), collected under the Italian funded FAO regional project, GTFS/INT/907/ITA (as described previously [20–22]), were used to evaluate the assays for the FMDV serotypes O, A and Asia-1. The serotype and subtypes of these samples had been identified using phylogenetic analyses of the VP1 coding sequences of these viruses. These sequences have been deposited in GenBank and the accession numbers are listed (see S1 Table and [20–22,36]). In addition, the serotype of a few samples had been determined using the antigen-ELISA. One sample (2953), that was positive in the pan-FMDV RT–qPCR assays, had not been serotyped, by sequencing, due to inadequate yield of product in the RT-PCR. Clinical samples collected from cases of FMD from Iran (2010) (unpublished results) and Bulgaria (2011) [5] were also included in the analyses. Details of the all the samples are shown in the S1 Table.

### Sample preparation

#### Epithelial samples

The epithelial samples were homogenized in RLT buffer (Qiagen, Hilden, Germany) prior to RNA extraction as described previously [20]. Briefly, the epithelial samples were processed by mixing with disruption beads and 1 ml of RLT buffer in a homogenizer (FastPrep, FP120 Thermo Electron Corporation) for 30 seconds at a setting of 6.5 m/s. After centrifugation of the homogenate at 14462 × *g* for 10 minutes (min), the supernatants were passed through QIA Shredder Mini Spin Columns (Qiagen). The filtrates were used for RNA extraction.

#### Oral swab samples

The oral swab samples, preserved in RLT buffer, were thawed, vortexed and then centrifuged at 664 × *g* for 10 min. The supernatants were used for RNA extraction.

### RNA extraction and cDNA synthesis

Total RNA was extracted from the samples either using manual (QIAamp RNA Blood Mini Kit) or robotic (MagNA Pure) protocols as described elsewhere [23]. In each case, the RNA was eluted in 50 μl of water.

The cDNA was synthesised by adding 7 μl of random hexamer primers (50 ng/μl) to 20 μl of template RNA and 8 μl water. The samples were heated to 65°C for 10 min, cooled on ice for 2 min and then transferred into tubes containing Ready-to-Go beads (GE Healthcare Life Sciences), vortexed and incubated at 40°C for 60 min followed by incubation at 90°C for 5 min to heat inactivate the reverse transcriptase. Water was added to the cDNA to give a volume of 200 μl to provide sufficient test material for each of the assays.

### Pan-FMDV and serotype-specific RT-qPCR assays

The cDNA was used in RT-qPCR assays using the serotype-specific primers/probes sets designed in this study and run in parallel with the primers/probe set described by Reid and colleagues [33] and the pan-FMDV primers/probe sets targeting the 3D coding region [10] and the 5′-UTR [12]. The reactions were performed in a MX4000 thermal cycler (Stratagene, The Netherlands) using 50 cycles of amplification with the following programme: 50°C for 2 min for UNG digestion, 1 cycle; denaturation at 95°C for 10 min, 1 cycle; 95°C for 15 sec, 60°C (or 55°C for Asia-1 assay) 1 min, 50 cycles. The C_T_ values obtained with pan-FMDV assays (3D and 5'-UTR) and the serotype-specific assays were compared. The primers/probe sets of the serotype-specific assays were aligned (using MEGA6 software) with the VP1 coding region nucleotide sequences of the samples that were not efficiently amplified in the assays or showed a clear (>4) difference in C_T_ values between the different assays.

## Results

### Diagnostic sensitivity and specificity of serotype-specific RT-qPCR assays

RNA samples derived from known cases of FMD were assayed using the well-established diagnostic RT-qPCR assays [10,12]. These assays included the pan-FMDV assays, targeting part of the 3D coding region and the 5'-UTR within the FMDV genome, plus the serotype-specific assays described previously [33] that target the VP1 coding regions of FMDV serotypes circulating in the Middle East. In addition, the same samples were assayed using the multiple primers/probe sets designed in this study to recognize the coding sequences for the VP1 proteins of FMDV serotypes O, A and Asia-1 currently circulating in West Eurasia. The data generated are summarized in Table 2. The pan-FMDV assays confirmed the presence of FMDV RNA in all the 59 samples tested.

**Table 2:** Summary of the RT-qPCR results using serotype O-, A- and Asia-1-specific and pan-FMDV 5'-UTR and 3D assays.

Serotype	5'-UTR assay	3D assay	O (Reid et al. [33] assay)	O (“2^nd^ generation” assay)	A (Reid et al. [33] assay)	A (“2^nd^ generation” assay)	Asia-1 (Reid et al. [33] assay)	Asia-1 (“2^nd^ generation” assay)
O	24*/24	24/24	23**/24	24/24	1/24	0/24	0/24	0/24
A	25/25	25/25	0/25	0/25	21/25***	23/25***	1/25***	1/25**
Asia-1	10/10	10/10	0/10	0/10	0/10***	1/10***	10/10	10/10

* two samples had high Ct values (38.9 and 37.9)

** two samples had high Ct values (38.1 and 38.9)

*** includes sample positive for both serotypes A and Asia-1

#### Use of the “first generation” serotype specific assays

**Fig 1:**
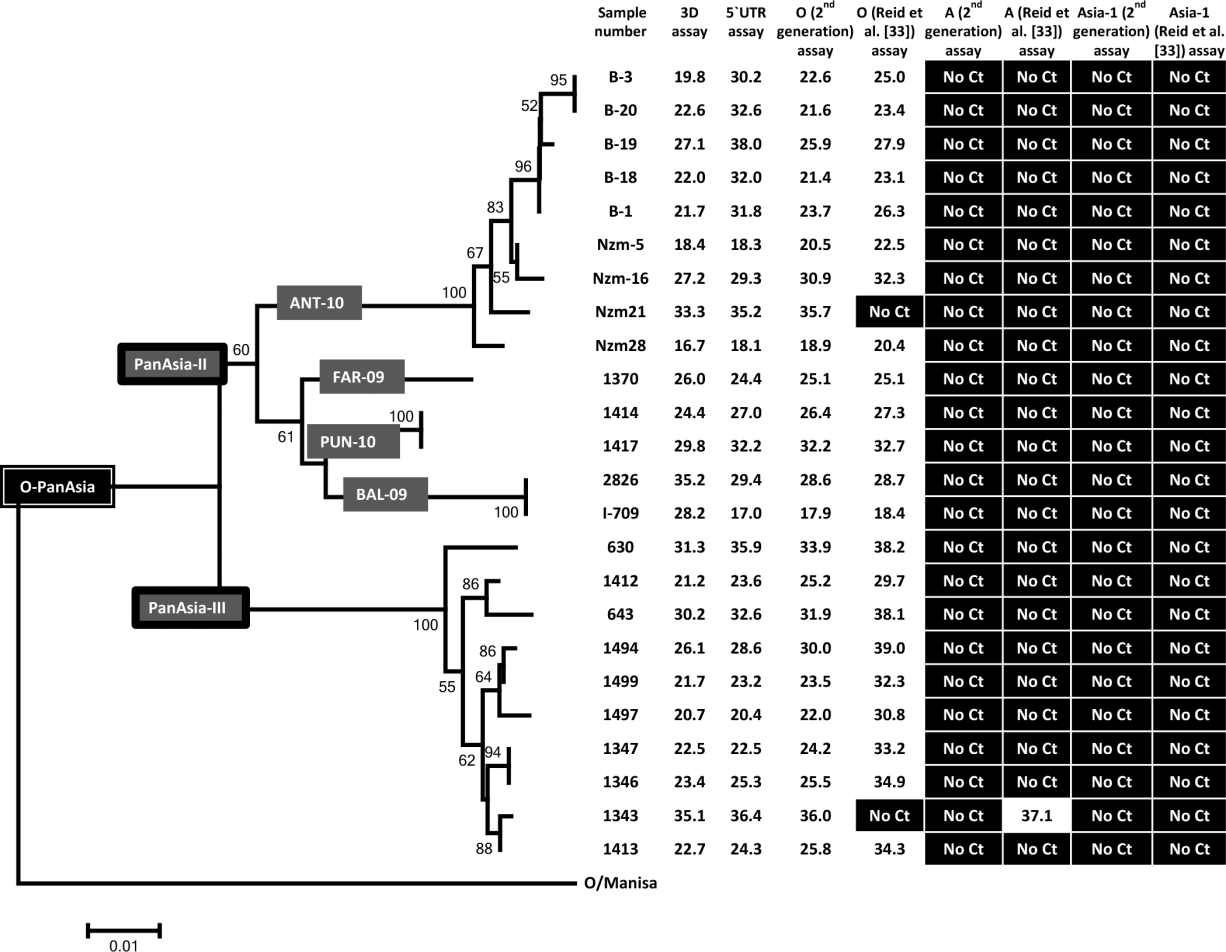
Diagnostic sensitivity and specificity of FMDV serotype O specific RT-qPCR assays. The C_T_ values obtained in the different RT-qPCR assays for the indicated serotype O virus samples are shown. The genetic relationships between the various strains from West Eurasia, based on VP1 coding sequences, are also indicated.

The “first generation” serotype O-specific RT-qPCR assay described previously by Reid and colleagues [33] were able to identify FMDV serotype O in 22 out of 24 samples that were known to be positive for serotype O FMDV. These samples contained various different virus strains within O-PanAsia i.e. O-PanAsia-II^ANT-10^, O-PanAsia-II^FAR-09^, O-PanAsia-II^PUN-10^, O-PanAsia-II^BAL-09^ and O-PanAsia-III (Fig 1). Two samples could not be detected by these serotype O-specific assays; these belonged to the O-PanAsia-II^ANT-10^ sublineage (sample No. Nzm21) and O-PanAsia-III (sample No. 1343) lineage. Indeed, the latter sample was mis-classified as belonging to serotype A using this assay. However, the assay was able to identify one sample (B-17), obtained from serotype O FMDV outbreaks in Bulgaria in 2011, as serotype O that had been identified as positive in the pan-FMDV RT-qPCR but its serotype was unknown. Using this collection of samples, the diagnostic sensitivity of the “first generation” serotype O specific assay was found to be 91.7% [95% CI = 80.6–100%] and the specificity was 97.1% [95% CI = 91.6–100%].

**Fig 2:**
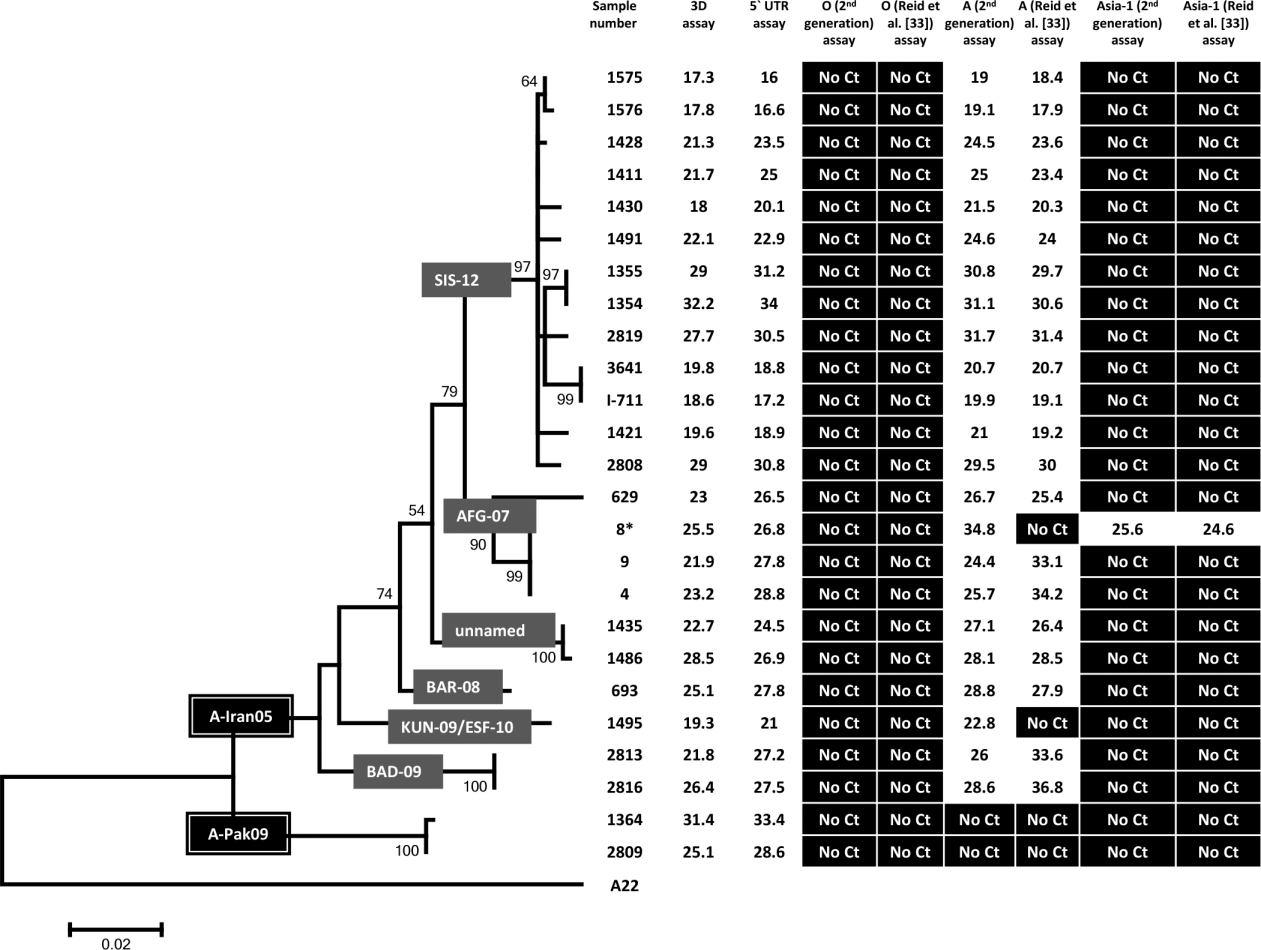
Diagnostic sensitivity and specificity of FMDV serotype A-specific RT-qPCR assays. The C_T_ values obtained in the different RT-qPCR assays for the indicated serotype A virus samples are shown. The genetic relationships between the various strains from West Eurasia, based on VP1 coding sequences, are also indicated. Note, sample 8 is marked with an * to indicate this sample contained two different serotypes of FMDV.

**Fig 3:**
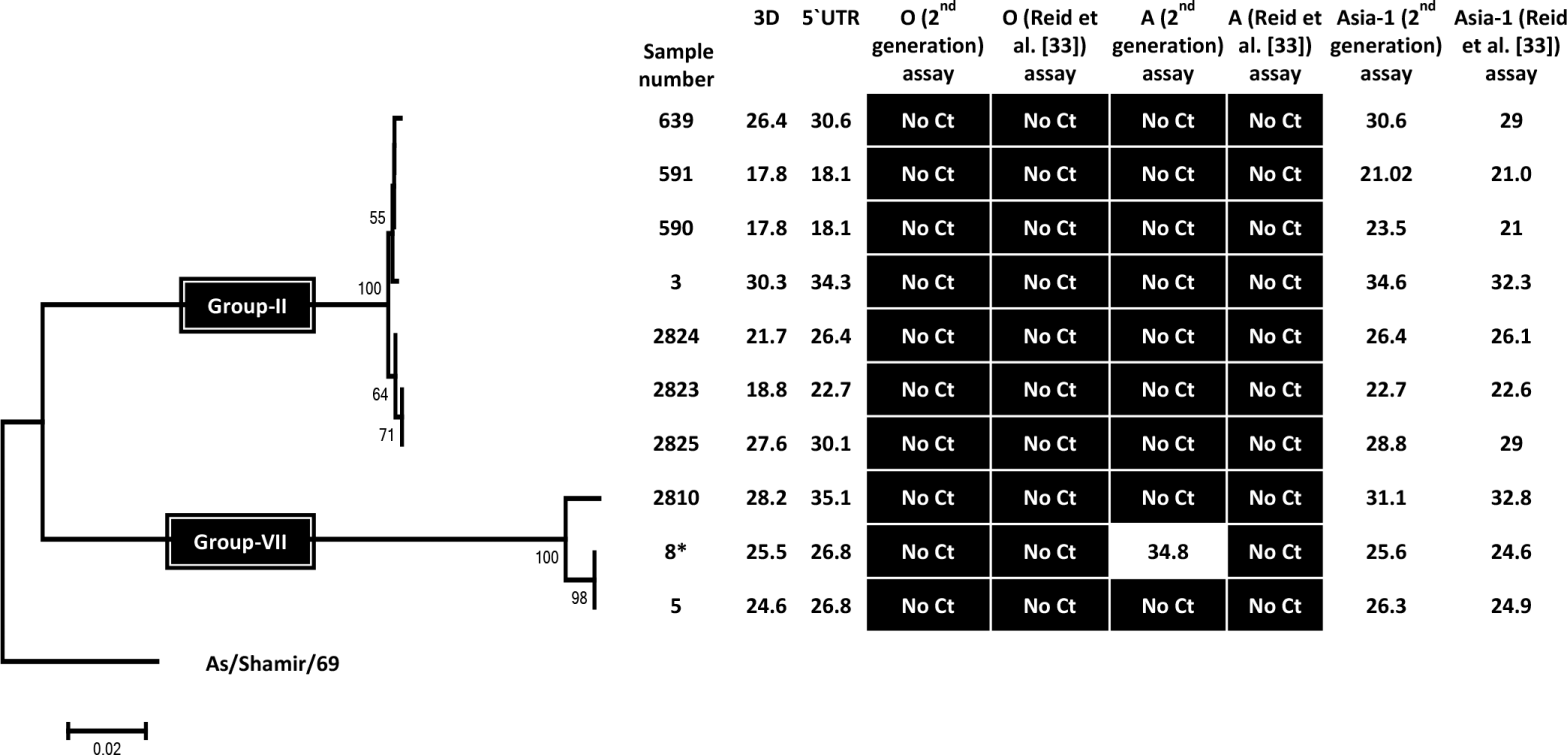
Diagnostic sensitivity and specificity of FMDV serotype Asia-1-specific RT-qPCR assay. The C_T_ values obtained in the different RT-qPCR assays for the indicated serotype Asia-1 virus samples are shown. The genetic relationships between the various strains from West Eurasia, based on VP1 coding sequences, are also indicated. Note, sample 8 is marked with an * to indicate this sample contained two different serotypes of FMDV.

The Reid et al. [33] serotype A-specific assay correctly detected and identified serotype A FMDV in 21 out of 25 serotype A FMDV positive samples. Among the four samples which were not detected, two belong to the A-Iran05 lineage while two others belong to a different lineage, designated as the A-Pak09 lineage [22] (Fig 2). The two undetected FMDVs belonging to the A-Iran05 lineage were members of the A-Iran05^AFG-07^ and A-Iran05^KUN-09^ sublineages (note this sub-lineage has also been termed A-Iran05^ESF-10^ by the WRL-FMD). The diagnostic sensitivity of this assay was calculated to be 84% [95% CI = 69.6–98.4%] and the specificity was 97.1% [95% CI = 91.6–100%]. The “first generation” serotype Asia-1 specific assay successfully detected all the 10 serotype Asia-1 positive samples belonging to two different Groups, i.e. Group II and Group VII/Sindh-08 (Fig 3), thus showing 100% diagnostic sensitivity and specificity.

#### Use of “second generation” serotype-specific assays

The “second generation” serotype O-specific assay described here successfully detected and identified all the 24 serotype O FMDVs tested. As with the earlier serotype O-specific assay [24], the new assay was able to assign serotype identity to one sample (B-17) that had been identified as positive in the pan-FMDV RT-qPCR but whose serotype was unknown. Both the diagnostic sensitivity and specificity of this assay was found to be 100%. In addition, of the 25 serotype A FMDV positive samples, the “second generation” serotype A-specific assay detected 23 of them but could not detect two. These two samples belonged to the A-Pak09 lineage (Fig 2) and were not detected by the Reid et al. [33] assays either (see above). The diagnostic sensitivity and specificity of this assay was found to be 92% [95% CI = 81.4–100%] and 100%, respectively. The new serotype Asia-1-specific assay correctly classified all the 10 serotype Asia-1 FMDVs as positive for this serotype with no cross reactivity with other serotypes, showing 100% diagnostic sensitivity and specificity.

As indicated above, one serotype O positive sample (1343) tested positive for serotype A in the Reid et al. [33] serotype A specific assay but negative with the serotype O-specific set. This sample was, however, identified as positive only for serotype O using the “second generation” serotype-specific assays (Fig 1), consistent with the sequence determination. In principle, it is possible, although unlikely, that this sample contained a mixture of serotype O and A viruses and that the new serotype A-specific assay failed to detect the presence of the serotype A virus while the Reid et al. [33] serotype O-specific assay failed to detect the serotype O FMDV. In summary, no apparent cross-reactivity was observed when the “second generation” serotype-specific assays were tested on samples containing each of the serotype O, A and Asia-1 FMDVs. Sample No. 8, which contained both serotypes A and Asia-1 [21,22], tested positive for both serotypes within the “second generation” serotype-specific assay as expected. This sample tested positive for serotype Asia-1 FMDV with the Reid et al. [33] serotype-specific assay but not for serotype A. Both the “first” and “second generation” serotype-specific assays were successful in assigning the serotype identity to sample No. 2953 as serotype Asia-1, that was also positive in both the pan-FMDV 3D and 5'-UTR assays but could not be sequenced (and hence serotyped) due to the low yield of the conventional RT-PCR product.

### Comparison of cycle threshold (C_T_) values obtained in the different RT-qPCR assays

**Fig 4:**
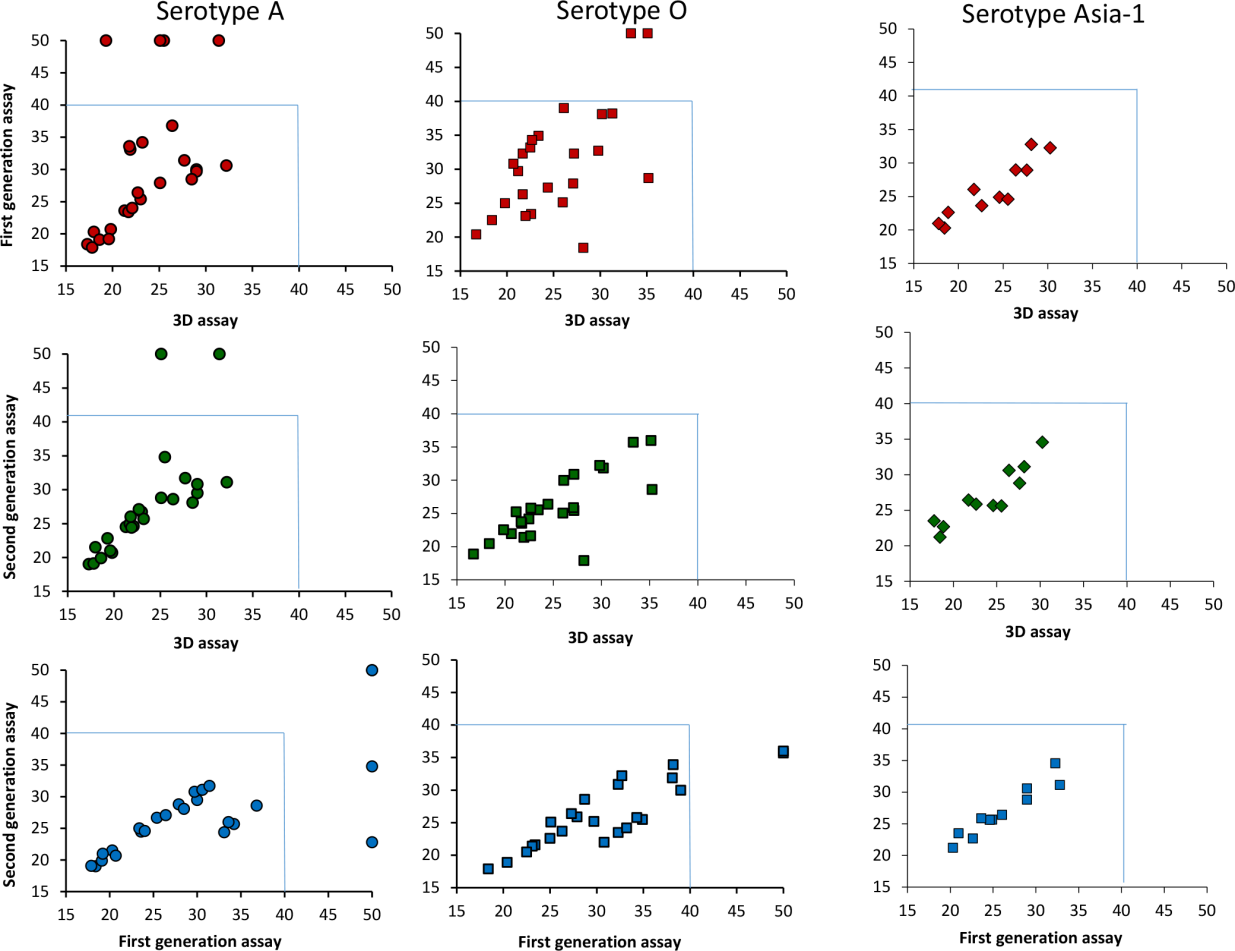
Comparison of cycle threshold (C_T_) values obtained in the different RT-qPCR assays. C_T_ values obtained with the pan-FMDV 3D assay, the Reid et al. [33] assays and “second generation” serotype-specific assays described here for serotypes A, O and Asia-1 viruses are shown.

A comparison between the cycle threshold (C_T_) values for the pan-FMDV assays, the Reid et al. [33] serotype-specific assays and the “second generation” serotype-specific assay is shown in Fig 4. Similar C_T_ values were obtained for the samples positive in the pan-FMDV assays and the “second generation” assays. In contrast, C_T_ values obtained with pan-FMDV 3D assay and the Reid et al. [33] assays show some differences particularly for the serotype O and A FMDVs that were tested. Lower C_T_ values were obtained in the “second generation” assay for 8 samples belonging to serotype O and for 4 serotype A FMDVs compared to the Reid et al. [33] assays (Figs 1 and 4), indicating that the new assays are more sensitive. Although no significant differences were noted in C_T_ values obtained between the two serotype-specific assays specific for the serotype Asia-1 FMDVs, clear differences can be noted in the slope of the amplification curves for the two assays (please see below for more detail).

### Nucleotide substitutions at primer/probe binding sites

**Fig 5:**
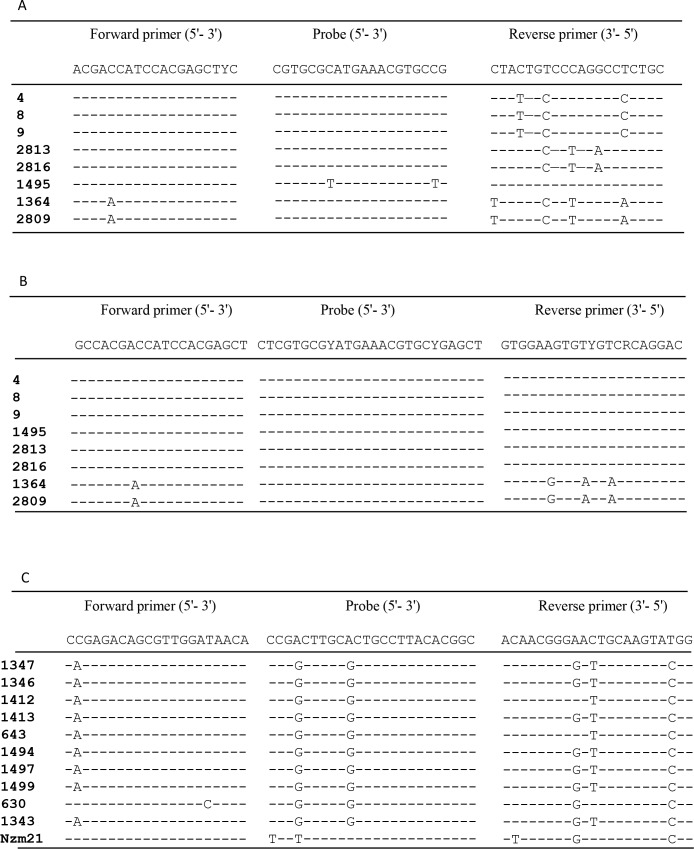
Comparison between nucleotide sequences of selected FMDV samples and the primers and probes. Panel (A) Sequences of serotype A FMDV samples indicating mismatches with primers and probe of the Reid et al. [33] serotype A-specific assay. Samples numbered 1495, 1364 and 2809 were not detected in the serotype A-specific assays described by Reid and colleagues [33]. Panel (B) Sequences of serotype A FMDVs indicating mismatches with primers and probe of the new serotype A-specific assay. Samples numbered 1364 and 2809 were not detected in the new assay. Panel (C) Nucleotide sequences of serotype O FMDV samples indicating mismatches with primer and probe sequences of the Reid et al. [33] serotype O-specific assay. The samples 1343 and Nzm21 were not detected in this assay.

The FMDV RNA samples which were not detected in either of the two serotype-specific assays or that showed a clear (>4) difference in the C_T_ values between these assays were investigated further. The primer/probe binding sites within the virus genomes were compared to the respective primer and probe sequences to identify nucleotide substitutions at these sites; these are shown in Fig 5. One nucleotide mismatch to the forward primer and four nucleotide mismatches to the reverse primer were noted in the corresponding binding sites within the VP1 coding region of the two samples (samples No. 1364 and 1809) which were not amplified in either the Reid et al. [33] (Fig 5A) or the “second generation” serotype A-specific assay (Fig 5B). As indicated above, these samples contained FMDVs that belong to the A-Pak09 cluster. Sample No. 1495, which was not detected in the Reid et al. [33] serotype A-specific assay, has two nucleotide mismatches in the probe binding site (Fig 5A). Similarly, the Reid et al. [33] serotype A-specific assay failed to identify serotype A within sample No. 8 (which actually contained both serotype A and Asia-1). Comparison of the sequences within the primers and probe binding sites of this sample revealed three nucleotide mismatches in the reverse primer of the first generation serotype A-specific assay (Fig 5A). Four serotype A positive samples, that showed clear difference in C_T_ values between the two serotype-specific assays, have three nucleotide mismatches in the reverse primer binding site in the Reid et al. [33] serotype A-specific assay (Fig 5B), which may explain the higher C_T_ values obtained.

Of the two serotype O positive samples that the Reid et al. [33] serotype O-specific assay did not detect, one (1343, belonging to the O-PanAsia-III lineage) had one nucleotide mismatch in the forward primer binding site, two mismatches in the probe binding site and three substitutions in the reverse primer binding site (Fig 5C). These mismatches may have reduced the efficiency of detection and it is noteworthy that the level of FMDV RNA in this sample was low as well (note the high C_T_ values obtained in the pan-FMDV assays). Two mismatches in the probe binding site and three mismatches in the reverse primer binding site were noted in the second FMDV (Nzm21, belonging to O-PanAsia-II^ANT-10^ sublineage) which was not amplified in the Reid et al. [33] serotype O-specific assay. Among the three mismatches in the reverse primer binding site, one substitution occurred in the second nucleotide from the 3´ end (Fig 5C) which may explain this failure in detection.

### Comparison of amplification curves of the serotype-specific RT-qPCR assays

**Fig 6:**
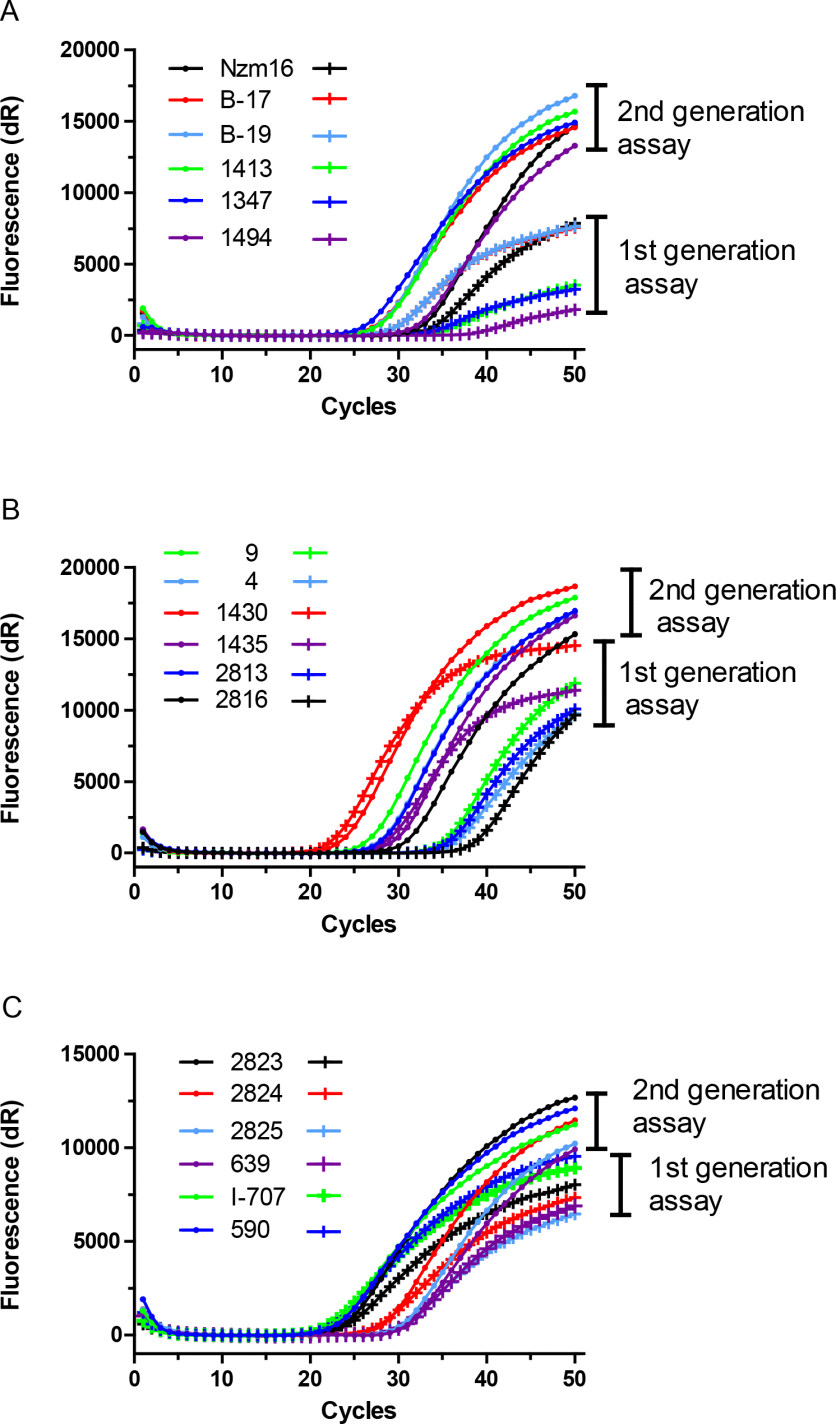
Comparison of amplification curves of the serotype-specific RT-qPCR assays. (A) Amplification curves for the six selected serotype O samples obtained with the 1^st^ generation assay [33] (+ symbols) and the 2^nd^ generation serotype O-specific assay described here (round symbols). (B) Amplification curves for the six selected serotype A samples obtained with the 1^st^ generation assay [33] (+ symbols) and the 2^nd^ generation serotype A-specific assay described here (round symbols). (C) Amplification curves for the six selected serotype Asia-1 samples obtained with the 1^st^ generation assay [33] (+ symbols) and the 2^nd^ generation serotype Asia-1-specific assay described here (round symbols).

A comparison of the amplification curves of selected samples between the Reid et al. [33] assays and the “second generation” serotype-specific assays individually for serotypes O, A and Asia-1 FMDVs is shown in Fig 6. Clear differences can be noted in the slope of amplification curves of the new (2^nd^ generation) and the Reid et al. [33] serotype O-specific assays (see Fig 6(A)). The amplification curves seen in the serotype A-specific assays were also more homogeneous in the new assay compared to the Reid et al. [33] serotype A-specific assay (see Fig 6(B)). Similarly, the amplification curves for serotype Asia-1 FMDVs were more consistent in shape in the new serotype Asia-1 specific assay and in many cases yielded higher fluorescence (dR) values compared to that of the Reid et al. [33] assay (Fig 6(C)).

## Discussion

Various techniques are in use for diagnosis of FMD including ELISA, virus isolation, conventional and real time RT-qPCR assays [1]. In order to control FMD outbreaks, an early and accurate identification of the causative FMDV is very important. In countries using a “stamping out” policy, simple detection of FMDV can be sufficient. Virus detection is usually carried out using pan-FMDV FMDV RT-qPCR assays that have been extensively evaluated for routine use [10–12,14]. Simple detection of FMDV using these assays is, however, not sufficient in countries where prophylactic vaccination is used to contain the disease as serotype identification is crucial to allow selection of the most appropriate vaccine, at least at the serotype level, as there is no cross-protection. Ideally the vaccine should be well matched to the outbreak virus but often there are practical limitations about this, e.g. in terms of vaccine availability, and vaccine matching studies or sequence determination can take some time.

Identification of conserved sequences as targets for primers and probes to specifically recognise all viruses within a particular serotype but without cross reactivity towards viruses of other serotypes is challenging. This is due to the high heterogeneity within the region of the FMDV genome (encoding the capsid proteins) that determines serotype and the lack of conserved sequences within all strains in individual serotypes worldwide. In order to overcome this issue, efforts have been made to detect viruses belonging to specific lineages within particular serotype(s) circulating in distinct geographical regions rather than across all strains globally [32–34]. This study builds on the use of this approach to develop serotype-specific RT-qPCR assays for detection and serotyping of FMDVs circulating in West Eurasia.

In the present study, primers and probe sets specific for serotypes O, A and Asia-1 FMDV were designed by individually aligning the VP1 coding sequences of serotypes O, A and Asia-1 FMDV circulating in West Eurasia. Comparison of the VP1 coding nucleotide sequences reveals a tendency for similar viruses to circulate in the same geographical area and analyses of these sequences have distributed FMDVs into seven different regional pools [37,38]. The primer and probe sets designed in this study effectively and specifically detected genomes belonging to a variety of different FMDV sublineages of O-PanAsia (see Fig 1), A-Iran05 (see Fig 2) and Asia-1 strains (see Fig 3) as intended. Two samples of serotype A FMDV that were not detected belong to a distinct lineage, designated as A-Pak09 [22]. The serotype-specific assays described by Reid and colleagues [33] also failed to amplify these two samples and additionally failed to amplify some other FMDV positive samples either belonging to the A-Iran05 or the O-PanAsia lineages. The pan-FMDV assays do recognize these strains so it is apparent that FMDV is present. It should be possible to design assays specifically to detect this lineage if required. There is a continued need to adapt such assays to strains circulating within a region but clearly the assays described here recognize most of the viruses that have circulated recently within this region.

The new assays described here did not show any cross-reactivity with heterotypic serotypes of FMDV. However, some apparent cross-reactivity was noted in the assays described by Reid et al. [33] as one sample, identified as positive for serotype O FMDV also tested positive for serotype A. The clear detection of both serotype A and Asia-1 FMDV in one sample (sample 8) that indeed contained viruses of both serotypes (see [21,22]) reveals the utility and advantages of the RT-qPCR assays in identifying mixed infections over ELISA-based assays in which some cross-reactivity frequently occurs [1].

Cycle threshold (C_T_) values are not only used to determine the presence of FMDV RNA but they are also used to quantify the level of viral RNA in a sample. The C_T_ values obtained with the assays reported here were consistent with that of the pan-FMDV 3D or 5'-UTR assays. However, differences in C_T_ values were observed between the Reid et al. [33] assays and the new assays reported here; in some cases, lower C_T_ values were recorded with the new assays reported here compared to the Reid et al. [33] assays indicative of higher sensitivity.

In the TaqMan RT-qPCR assays, the amount of amplified DNA is measured after each cycle of amplification via dyes that generate fluorescent signals, the magnitude of which is proportional to the amount of the amplicon generated. The amplification curves, generated by plotting the fluorescence against the number of cycles, represent the accumulation of product over the duration of the reaction. Initially, the fluorescence signals increase exponentially with the number of thermal cycles. In general, the magnitude of the fluorescence signal was higher in the new assays for serotypes O, A and Asia-1 compared to that obtained in the Reid et al. [33] assays and the amplification curves were more consistent (Fig 6). It is apparent that a small number of nucleotide mismatches between the primers and probes can have a significant effect on the ability of the assays to detect the virus (see Fig 5); analogous observations have been reported previously [13].

Although the new serotype A-specific assays also failed to detect two samples positive for serotype A FMDVs (these do not belong to the A Iran05 lineage), the new serotype specific assays reported here have clear advantages over the Reid et al. [33] assays for samples collected within West Eurasia (e.g. see Fig 6) and thus may help in the early detection and typing of FMDV serotypes O, A and Asia-1 circulating there. Due to continuous changes in the FMDV RNA sequences, monitoring of the sequences, within circulating strains, at the primer and probe binding sites is required to ensure that the diagnostic sensitivity and specificity of these assays is maintained.

## Supporting Information

S1 Table Details of the FMDV positive samples used in this study. (DOCX)
