# Supplementary material for: Development and Characterization of Probe-Based Real Time Quantitative RT-PCR Assays for Detection and Serotyping of Foot-And-Mouth Disease Viruses Circulating in West Eurasia
Source: PLoS One. 2015 Aug 13;10(8):e0135559. doi: 10.1371/journal.pone.0135559 (PMC4535879; doi:10.1371/journal.pone.0135559)
Supplement: S1 Table — (DOCX) [file pone.0135559.s001.docx]

**S1 Table. Details of the FMDV positive samples used in this study**

| **Sample Number** | **Serotype** | **Reference Number** | **Year of collection** | **Country** | **Accession No.** | **Reference** |
| --- | --- | --- | --- | --- | --- | --- |
| 1343 | O | O/PUN/PAK/L1343/2008 | 2008 | Pakistan | HQ439207 | [20] |
| 1346 | O | O/PUN/PAK/L1346/2008 | 2008 | Pakistan | HQ439209 | [20] |
| 1347 | O | O/PUN/PAK/L1347/2008 | 2008 | Pakistan | HQ439206 | [20] |
| 1370 | O | O/PUN/PAK/L1370/2009 | 2009 | Pakistan | HQ439215 | [20] |
| 1412 | O | O/ISL/PAK/L1412/2009 | 2009 | Pakistan | HQ439214 | [20] |
| 1413 | O | O/ISL/PAK/L1413/2009 | 2009 | Pakistan | HQ439220 | [20] |
| 1414 | O | O/NWF/PAK/L1414/2009 | 2009 | Pakistan | HQ439212 | [20] |
| 1417 | O | O/NWF/PAK/L1417/2009 | 2009 | Pakistan | HQ439217 | [20] |
| 630 | O | O/KUN/AFG/L630/2009 | 2009 | Afghanistan | HQ439232 | [20] |
| 643 | O | O/KAB/AFG/L643/2009 | 2009 | Afghanistan | HQ439233 | [20] |
| 1494 | O | O/BAG/AFG/L1494/2009 | 2009 | Afghanistan | HQ439234 | [20] |
| 1497 | O | O/KUN/AFG/L1497/2009 | 2009 | Afghanistan | HQ439236 | [20] |
| 1499 | O | O/JAW/AFG/L1499/2009 | 2009 | Afghanistan | HQ439237 | [20] |
| 2826 | O | O/KAB/AFG/L2826/2009 | 2009 | Afghanistan | HQ439238 | [20] |
| I-709 | O | O/KAB/AFG/I-709/2009 | Rescued virus |  | --- | unpublished |
| B-1 | O | 12LPN1 | 2011 | Bulgaria | JX066664 | [37] |
| B-3 | O | 12LPN3 | 2011 | Bulgaria | JX066665 | [37] |
| B-18 | O | O/BG/L18/2011 | 2011 | Bulgaria | --- | unpublished |
| B-19 | O | O/BG/L19/2011 | 2011 | Bulgaria | --- | unpublished |
| B-20 | O | O/BG/L20/2011 | 2010 | Bulgaria | --- | unpublished |
| Nzm-5 | O | O/IRN/L5/2010 | 2010 | Iran | --- | unpublished |
| Nzm-16 | O | O/IRN/L16/2010 | 2010 | Iran | --- | unpublished |
| Nzm-21 | O | O/IRN/L21/2010 | 2010 | Iran | --- | unpublished |
| Nzm-28 | O | O/IRN/L28/2010 | 2010 | Iran | --- | unpublished |
| 4 | A | A/SIN/PAK/L4/2008 | 2008 | Pakistan | HQ439239 | [22] |
| 9 | A | A/SIN/PAK/L9/2008 | 2008 | Pakistan | HQ439243 | [22] |
| 629 | A | A/KUN/AFG/L629/2009 | 2009 | Afghanistan | HQ439261 | [22] |
| 693 | A | A/SIN/PAK/L693/2009 | 2009 | Pakistan | HQ439246 | [22] |
| 1354 | A | A/PUN/PAK/L1354/2009 | 2009 | Pakistan | HQ439251 | [22] |
| 1355 | A | A/PUN/PAK/L1355/2009 | 2009 | Pakistan | HQ439252 | [22] |
| 1364 | A | A/PUN/PAK/L1364/2009 | 2009 | Pakistan | HQ439253 | [22] |
| 1411 | A | A/ISL/PAK/L1411/2009 | 2009 | Pakistan | HQ439256 | [22] |
| 2808 | A | A/PUN/PAK/L2808/2009 | 2009 | Pakistan | HQ439257 | [22] |
| 2809 | A | A/PUN/PAK/L2809/2009 | 2009 | Pakistan | HQ43925 | [22] |
| 1421 | A | A/TAK/AFG/L1421/2009 | 2009 | Afghanistan | HQ439265 | [22] |
| 1428 | A | A/PAN/AFG/L1428/2009 | 2009 | Afghanistan | HQ439269 | [22] |
| 1430 | A | A/BAL/AFG/L1430/2009 | 2009 | Afghanistan | HQ439270 | [22] |
| 1435 | A | A/SAR/AFG/L1435/2009 | 2009 | Afghanistan | HQ439274 | [22] |
| 1486 | A | A/HIR/AFG/L1486/2009 | 2009 | Afghanistan | HQ439283 | [22] |
| 1491 | A | A/KAP/AFG/L1491/2009 | 2009 | Afghanistan | HQ439284 | [22] |
| 1495 | A | A/KUN/AFG/L1495/2009 | 2009 | Afghanistan | HQ439287 | [22] |
| 1575 | A | A/HIR/AFG/L1575/2009 | Rescued virus |  | HQ439291 | [22] |
| 1576 | A | A/HIR/AFG/L1576/2009 | Rescued virus |  | HQ439292 | [22] |
| 2813 | A | A/BAD/AFG/L2813/2009 | 2009 | Afghanistan | HQ439295 | [22] |
| 2816 | A | A/BAD/AFG/L2816/2009 | 2009 | Afghanistan | HQ439298 | [22] |
| 2819 | A | A/BAD/AFG/L2819/2009 | 2009 | Afghanistan | HQ439301 | [22] |
| 3641 | A | A/PAN/AFG/L3641/2009 | 2009 | Afghanistan | HQ439309 | [22] |
| I-711 | A | A/PAN/AFG/I-711/2009 | Rescued virus |  | --- | unpublished |
| 8* | A/Asia-1 | A/SIN/PAK/L8/2008  As/ SIN/PAK/L8/2008 | 2008 | Pakistan | HQ439242  HQ439188 | [22]  [21] |
| 3 | Asia-1 | As/BAM/AFG/L3/2009 | 2009 | Afghanistan | HQ439196 | [21] |
| 5 | Asia-1 | As/SIN/PAK/L5/2008 | 2008 | Pakistan | HQ439187 | [21] |
| 590 | Asia-1 | As/BAM/AFG/L590/2009 | Rescued virus |  | HQ113233 | [36] |
| 591 | Asia-1 | As/BAM/AFG/L591/2009 | Rescued virus |  | HQ439199 | [21] |
| 639 | Asia-1 | As/BAM/AFG/L639/2009 | 2009 | Afghanistan | HQ439198 | [21] |
| 2810 | Asia-1 | As/SIN/PAK/L2810/2009 | 2009 | Pakistan | HQ43918 | [21] |
| 2823 | Asia-1 | As/BAM/AFG/L2823/2009 | 2009 | Afghanistan | HQ439203 | [21] |
| 2824 | Asia-1 | As/BAM/AFG/L2824/2009 | 2009 | Afghanistan | HQ439204 | [21] |
| 2825 | Asia-1 | As/BAM/AFG/L2825/2009 | 2009 | Afghanistan | HQ439205 | [21] |
| I-707 | Asia-1 | --- | Rescued virus |  | --- |  |
| 2953 |  | --- | 2009 | Pakistan | --- |  |
| B-17 |  | --- | 2011 | Bulgaria | --- |  |

* positive for both serotypes A and Asia-1 FMD
